# Redundant causation from a sufficient cause perspective

**DOI:** 10.1186/1742-5573-7-5

**Published:** 2010-08-02

**Authors:** Nicolle M Gatto, Ulka B Campbell

**Affiliations:** 1 Department of Epidemiology, Mailman School of Public Health, Columbia University, New York, NY 10032 USA; 2 Epidemiology, Worldwide Safety Strategy, Pfizer Inc, New York, NY 10017 USA

## Abstract

Sufficient causes of disease are redundant when an individual acquires the components of two or more sufficient causes. In this circumstance, the individual still would have become diseased even if one of the sufficient causes had not been acquired. In the context of a study, when any individuals acquire components of more than one sufficient cause over the observation period, the etiologic effect of the exposure (defined as the absolute or relative difference between the proportion of the exposed who develop the disease by the end of the study period and the proportion of those individuals who would have developed the disease at the moment they did even in the absence of the exposure) may be underestimated. Even in the absence of confounding and bias, the observed effect estimate represents only a subset of the etiologic effect. This underestimation occurs regardless of the measure of effect used.

To some extent, redundancy of sufficient causes is always present, and under some circumstances, it may make a true cause of disease appear to be not causal. This problem is particularly relevant when the researcher's goal is to characterize the universe of sufficient causes of the disease, identify risk factors for targeted interventions, or construct causal diagrams. In this paper, we use the sufficient component cause model and the disease response type framework to show how redundant causation arises and the factors that determine the extent of its impact on epidemiologic effect measures.

## Introduction

Sufficient causes of disease are redundant when an individual acquires the components of two or more sufficient causes (SCs), so that even if one of the SCs had not been acquired, the individual still would have become diseased. Redundancy occurs because disease can result from one of multiple (sufficient) causes, as affirmed by the sufficient component cause model used in epidemiology. When any individuals under study have redundant SCs, the etiologic effect of the exposure may be underestimated. The etiologic effect is the absolute or relative difference between the proportion of the exposed who develop the disease by the end of the study period and the proportion of those individuals who would have developed the disease at the moment they did even in the absence of the exposure [1-3]. This occurs in the best-case scenario when confounding and bias are absent and regardless of the effect measure used. To some extent, individuals with redundant SCs are always present, and under some circumstances, redundancy may make a true cause of disease appear to be not causal. This problem is particularly relevant when the researcher's goal is to characterize the universe of SCs of the disease, identify risk factors for targeted interventions, or construct causal diagrams.

The idea that causes can be redundant has been discussed extensively in philosophic and legal literature on causation. Our perspective is that as epidemiologists, our goal is to identify all causes of disease regardless of when, how or how often they act. Redundancy of SCs of disease presents a barrier to this goal. This issue has been conceptually described by Mackie in the context of philosophy [4] and acknowledged by Rothman in his seminal SC paper [5]. Mackie used illustrative conceptual examples to describe redundancy of SCs and how it arises, but did not connect these to the impact on effect measures [4]. In their work on the definition and interpretation of attributable fractions and probability of causation, Greenland, Robins and Beyea analytically (through mathematical formula, and conceptual and numeric examples) demonstrated the impact of redundant causation on effect measures used in epidemiology [1-3,6,7]. In the most recent version of Modern Epidemiology, Greenland, Rothman and Lash described how redundancy arises using a simple SC example and noted its implication for estimating an etiologic effect ([8]).

Though a great deal of this work was published over 20 years ago, we suspect that most epidemiologists don't appreciate the potential impact of redundant SCs. It is rarely discussed in epidemiologic methods and to our knowledge, has not been discussed in any published applications. Yet, we believe redundancy has important implications for etiologic research and causal thinking, and perhaps should be carefully considered by epidemiologists as are confounding and bias.

While the work of Mackie [4] and Greenland and Robins [1-3,7] provide the backbone of our understanding of redundancy of SCs (and should be read for a full understanding of this phenomenon), it is not our intention to review their work. Rather, we hope to make this discussion more comprehensive and more accessible to epidemiologists by reframing and connecting the phenomenon described by Mackie to Greenland and Robins' analyses. To do this, we will: (1) use SCs to describe how redundancy of SCs arises and the factors that influence the proportion of redundant individuals in a population, (2) adapt the disease response type methodology (which has been used in epidemiology in various forms to explicate the effects of confounding [9], mediation [10-12] and effect modification [13-16]) to show how redundancy of SCs impacts epidemiologic effect measures, and (3) use a simple numeric example to show how the relative effect estimates calculated in epidemiologic studies underestimate the full exposure effect, potentially obscuring causal identification.

## Terminology

In published philosophic, legal and epidemiologic literature, and through our personal interactions, we have encountered several names for the situation in which an individual acquires more than one cause of disease. These names include over-determination, pre-emption, trumping, double-jeopardy, over-lap bias, and accelerated occurrence (For example, see [4,7,17]). In addition, this situation is related to several terms used in discussions of interaction (e.g. parallelism, competition and cooperative-competitive action) [13,18]. However, we find these terms to be limited because they are used narrowly to describe particular ways that redundancy of SCs can arise, are uncommonly used, or are undocumented in the literature. Thus, we use "redundancy of sufficient causes" as a descriptive umbrella term to encompass all sub-types of redundancy. The term "redundant causation" is commonly used in philosophic discussions of causation. For example, Lewis (page 193, [19]) stated:

"Suppose we have two events *c*_1 _and *c*_2_, and another event *e *distinct from both of them; and in actuality all three occur; and if either one of *c*_1 _or *c*_2 _had occurred without the other, then also *e *would have occurred; but if neither *c*_1 _or *c*_2 _had occurred, then *e *would not have occurred. Then I shall say that *c*_1 _and *c*_2 _are *redundant causes *of *e*."

Going forward, we will use the term "redundancy" for simplicity. However, we will note when we first describe a particular type of redundancy that has a documented name. Our intention is not to coin a new term, but rather to simplify our discussion of this phenomenon.

## The sufficient-component cause model

**Figure 1:**
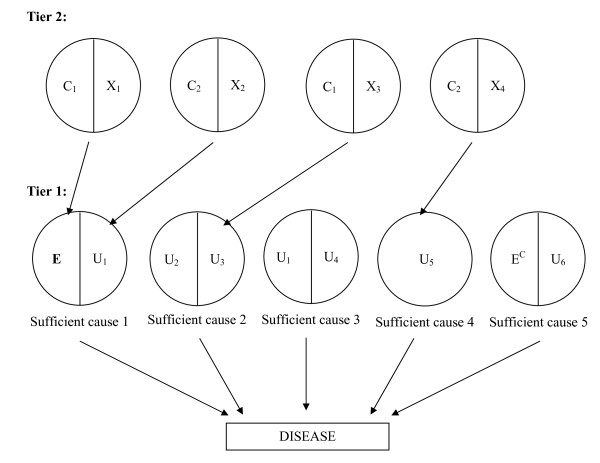
**Sufficient component causal (SCC) model with five sufficient causes of disease**. Disease may result from: (1) the presence of both E and U_1_; or (2) the presence of both U_2 _and U_3_; or (3) the presence of both U_1 _and U_4_; or (4) the presence of U_5_; or (5) the absence of E (i.e. the complement of E, E^C^) and the presence of U_6_.

Modern epidemiology is fundamentally based on the notion that for most diseases, an individual can develop a disease from one of many possible SCs, each of which consists of several components working together. This model of disease causation, known as the sufficient component cause (SCC) model [4,5], stipulates "constellations" of components. It states that although none of the components in any given constellation can cause disease by itself, each makes a non-redundant, necessary contribution to complete a causal mechanism. Thus, each component is a cause of disease; the SSC model specifies the circumstances in which each causal component acts. A constellation of components that is minimally sufficient to cause disease is termed a SC; SCs are often depicted as "causal pies". As an example, Figure 1: Tier 1, depicts five SCs of disease. An individual may develop the disease by: (1) acquiring both E and U_1_; or (2) acquiring both U_2 _and U_3_; or (3) acquiring both U_1 _and U_4_; or (4) acquiring U_5 _alone; or (5) not acquiring E and acquiring U_6 _(see endnote 1).

In our work, we conceptualize an expanded SCC model, in which tiers of SCs are connected to specify how relationships arise [20]. In these SCC models, we include all SCs of the disease (e.g. Figure 1: Tier 1), as well as any SC that contributes to an association between any causal components of the disease (e.g. Figure 1: Tier 2). Our approach is based on the premise that associations between any two components arise either because one component causes the other or because they are both caused by a third component. This premise is consistent with the definition of a causal directed acyclic graph (DAG), a type of causal diagram in which an arrow between two variables represents a causal effect and all common causes of each pair of variables are specified [21].

We create the simplest (i.e. most minimal) causal model to address the question at hand by extending the principles of DAGs and the SSC model. Thus, if there are no associations between the components of the SCs of disease, only one tier of SCs would be shown. In Figure 1, SCs 1 to 5 are labeled Tier 1 causes. We also show four Tier 2 causes. E is caused by acquiring both C_1 _and X_1_, component U_1 _is caused by acquiring both C_2 _and X_2_, component U_3 _is caused by acquiring both C_1 _and X_3 _and component U_5 _is caused by acquiring both C_2 _and X_4_. In this example, an association between E and U_3 _arises from a shared component cause, C_1_. To reflect that C_1 _is not sufficient to cause E and U_3_, we include X_1 _and X_3 _as its causal partners; C_1 _requires X_1 _to cause E and requires X_3 _to cause U_3_. Likewise, an association between U_1 _and U_5 _arises from a shared component cause, C_2_. To reflect that C_2 _is not sufficient to cause U_1 _and U_5_, we include X_2 _and X_4 _as its causal partners; C_2 _requires X_2 _to cause U_1 _and requires X_4 _to cause U_5_. Note that because C_1 _leads to an association between the exposure and a component cause of disease that does not work with the exposure, C_1 _causes confounding; C_1 _is a common cause of disease (via U_3_) and exposure, thus causing confounding. While C_2 _leads to an association between U_2 _and U_5_, C_2 _does not cause confounding. Rather, there is an association between the causal partner of the exposure and a component cause that does not work with the exposure. While this type of association does not bias effect estimates, it has important implications for redundancy that we discuss later.

**Figure 2:**
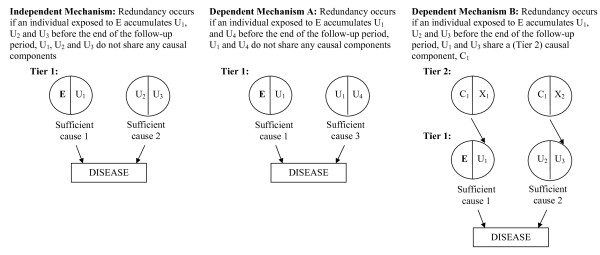
**How redundancy arises from the perspective of the Sufficient Component Cause model (E is the exposure of interest)**.

Redundancy must be described with respect to a causal component of interest, i.e. the exposure of interest. We will refer to the exposure of interest as "the exposure" and people who have acquired the exposure as "exposed" (and is reflected by "E" in Figures 1 and 2). Likewise, we refer to the disease of interest as "the disease" and people who have developed the disease as "diseased" or "cases". In addition, we refer to any other component causes of the SCs simply as "components" (e.g. in Figure 1: U_1_, U_2_, X_1_, X_2_, etc.). Below, we use SCC models to illustrate how redundancy arises. The consequences of redundancy will be discussed later in the paper when we discuss causal contrasts.

## How redundancy arises

Redundancy occurs at the level of the individual; by the end of some defined follow-up period, an individual has acquired more than one SC of the disease. From the perspective of the SCC model, redundancy can arise from independent or dependent processes, which is determined by whether an individual's probability of acquiring one SC is independent of or dependent on his/her probability of acquiring another SC. Redundancy can be considered "independent" when the causal partners of the exposure: (1) do not participate in any SC that does not include the exposure (i.e. the causal partners are "exclusive") *and *(2) do not share any common causes with components that do not work with the exposure. Because some individuals will acquire the components of more than 1 SC by chance, this type of redundancy is inevitable; it will always occur and is unavoidable (assuming there is more than one SC of a disease in a population). Redundancy can be considered "dependent" when at least one causal partner of the exposure: (1) is also a component in an SC that does not include the exposure (i.e. at least one causal partner is "non-exclusive") *or *(2) shares a common cause with a component that does not work with the exposure. This type of redundancy does not always occur, but rather is dependent on the causal model structure. Below, we describe examples of independent and dependent mechanisms of redundancy.

### Redundancy as a result of independent processes

In Figure 2: Independent Mechanism, redundancy arises from independent processes. By this we mean: 1) the causal partner of the exposure (U_1_) is exclusive (i.e. U_1 _works exclusively with E to cause disease,) and 2) U_1 _does not share an antecedent component cause with any other component cause of disease. Thus, there are no Tier 2 causes shown. Assuming these are the only SCs of the disease, redundancy occurs if any exposed individual acquires U_1_, U_2_, and U_3 _during the study period. Since there are no shared components between the SCs, one must be completed before the other [(E and U_1_) are acquired before (U_2 _and U_3_), or vice versa]. This type of redundancy has been described as pre-emption [17] and accelerated occurrence [7] (see endnote 2).

### Redundancy as a result of dependent processes

In Figure 2: Dependent Mechanism A, redundancy arises from dependent processes. The causal partner of the exposure (U_1_) is non-exclusive (i.e. U_1 _works with both E and U_4 _to cause disease). Assuming these are the only SCs of the disease, redundancy occurs if any exposed individual acquires U_1 _and U_4 _during the study period. Depending on the order of component acquisition, this could be pre-emption, or another type of redundancy known as over-determination [4]. If the shared component is not the last acquired component of both SCs of disease (i.e. if U_1 _is acquired before U_4_), then pre-emption occurs; one SC of disease must be fulfilled before the other. If the shared component (U_1_) is the last acquired component of both SCs of disease, then the SCs of disease are fulfilled simultaneously, which is over-determination.

In Figure 2: Dependent Mechanism B, redundancy also arises from dependent processes. Although the causal partner of the exposure (U_1_) is exclusive, U_1 _shares an antecedent component cause with a component cause of disease in another SC. Assuming these are the only SCs of the disease, redundancy occurs if any exposed individual acquires U_1_, U_2 _and U_3 _during the study period. Here, U_1 _and U_3 _share a Tier 2 common cause, C_1_. The causal partner of E shares a causal component with a component in an SC that does not include the exposure, leading to dependent redundancy. If C_1 _is not the last acquired component, then one SC will be fulfilled before the other (pre-emption). If an exposed individual has X_1_, X_2 _and U_2_, and the shared component (C_1_) is the last acquired component, then U_1 _and U_3 _are acquired simultaneously, and the SCs of disease are fulfilled simultaneously (over-determination).

## Determinants of the proportion of redundant individuals

Redundancy occurs at the level of the individual; an individual may or may not acquire more than 1 SC over a given time period. As we show below, two factors determine the proportion of redundant individuals in the population: (1) the distribution of the components in the population, and (2) the structure of the underlying SCC model.

### Distribution of components in the population

The proportion of redundant individuals in a population increases with the prevalences of the components that participate in the SCs of disease. For example, in the Independent Mechanism depicted see Figure 2, the likelihood of acquiring both SC1 and SC2 (for an exposed person) is equal to p(U_1_) * p(U_2_) * p(U_3_), where p(U_1_) is equal to the prevalence of component U_1_, etc. As the prevalence of any of these components increases, the proportion of individuals who acquire both SC1 and SC2 increases. For example, if p(U_1_), p(U_2_), p(U_3_) each equal 40%, then 6.4% of the population will acquire SC1 and SC2 and thus have redundant SCs. All else equal, if p(U_2_) increases to 60%, then 9.6% of the population will acquire SC1 and SC2 and have redundant SCs.

### Whether redundancy arises from independent or dependent mechanisms

In the SC underlying Dependent Mechanism A in Figure 2, the likelihood of acquiring both SC1 and SC3 (for an exposed person) is equal to p(U_1_) * p(U_4_). Compared with the Independent Mechanism, there are more individuals who acquire both SCs of disease because these SCs share a component. For example, if p(U_1_) and p(U_4_) are each equal to 40%, 16% of the population will acquire SC1 and SC3 and have redundant SCs. In the SC underlying Dependent Mechanism B in Figure 2, the proportion of redundant individuals (i.e. individuals who acquire both SC1 and SC2) is also greater than in the Independent Mechanism. For Dependent Mechanism B, the proportion of redundant individuals would be calculated using the prevalence of U_2 _(Tier 1 component), and the prevalences of C_1_, X_1_, and X_2 _(Tier 2 components).

## How redundancy obscures causal identification

In this paper, we use Rothman's definition of a cause - a component is considered to be a cause if it was necessary for the disease to occur at the moment it did in at least one person in the population [22]. In other words, a component is causal if it participated in the first fulfilled SC of disease for at least one person, whether or not this person would have become diseased at a later time by acquiring another SC. The following discussion assumes that our goal is to determine whether a particular component of interest (i.e. the exposure) meets this definition of a cause, and is restricted to the causal effect of the exposure in a group of people actually exposed. Ideally, we would like to compare the disease experience of each individual in a group of exposed people to the group consisting of their counterfactuals. This comparison between the risk of disease in the group of exposed individuals and the risk of disease in the same group of individuals under the condition of non-exposure is referred to as a *causal contrast*, which is the true but unobservable representation of the causal effect of the exposure in a given population at a given time (note: this is one of several causal contrasts) [23].

Causal contrasts are composed of two parts. The first part of our contrast of interest is the proportion of diseased people (i.e., risk of disease) among the exposed at the end of a specified time period. The second part is the proportion of diseased people among the exposed had they not been exposed (i.e., the counterfactual risk of disease). This causal contrast may refer to the full etiologic effect, or a subset of the full etiologic effect, i.e. the excess effect [3].

### Distinction between etiologic and excess causation under the SCC model

Greenland and Robins differentiated between two types of diseased individuals that are germane to our work - etiologic cases and excess cases [3]. An etiologic case is any diseased individual for whom the exposure of interest was a cause [1-3]. An excess case is any diseased individual for whom the disease would not have occurred by the end of the study period in the absence of the exposure. The difference may appear subtle. For an etiologic case, the exposure was necessary for disease to occur at the moment it occurred. For an excess case, the exposure was necessary for disease to occur at all. All excess cases are etiologic cases, but an etiologic case is only an excess case if the exposure was necessary for the disease to occur by the end of the study period [1-3].

**Figure 3:**
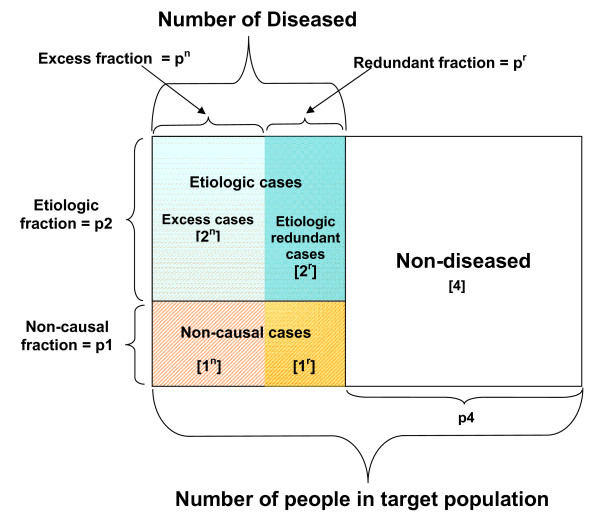
**Relationship among etiologic, excess, etiologic redundant cases and non-causal cases**. Where, 'p' means proportion, 'n' means non-redundant, and 'r' means redundant.

We can express Greenland's and Robin's analytic distinction between etiologic and excess cases in terms of the SCC model. An etiologic case is an individual who developed the disease from a SC that included the exposure of interest. An excess case is a type of etiologic case who does not later, over the course of the study, acquire any SC that excludes the exposure of interest (i.e. a non-redundant etiologic case). We can therefore split etiologic cases into excess and redundant cases. An etiologic redundant case is another type of etiologic case who later, over the course of the study, acquires at least one SC that excludes the exposure of interest. All etiologic cases are either excess or etiologic redundant cases. In contrast to an etiologic case, a non-causal case is an individual who developed the disease from a SC that excludes the exposure. The relationship among etiologic, excess, etiologic redundant and non-causal cases is illustrated in Figure 3.

The etiologic fraction, the proportion of cases for whom the exposure was a cause, is equal to the number of etiologic cases divided by the total number of diseased people at the end of the study [1-3]. We can subdivide the etiologic fraction into the excess fraction and etiologic redundant fraction. The excess fraction is the subset of the etiologic fraction that is non-redundant and is equal to the number of excess cases divided by the total number of diseased [1-3]. The difference between the etiologic fraction and the excess fraction is the etiologic redundant fraction, i.e. the number of etiologic redundant cases (i.e. people who got the disease from the exposure but would have gotten it from another SC by the end of the study period) divided by the total number of diseased. The non-causal fraction, the proportion of cases for whom the exposure was not a cause, is equal to the number of non-causal cases divided by the total number of diseased people at the end of the study. The relationship among etiologic, excess, etiologic redundant and non-causal fractions is also illustrated in Figure 3.

Below, we use disease response types to quantify these fractions, as well as the etiologic and excess causal effects and the discrepancy between them. We use the response types as a tool because they are simple and descriptive.

### Further specification of response types

In their 1986 paper, Greenland and Robins [9] described four possible disease responses to a given exposure: doomed, susceptible-causative, susceptible-preventive and immune. Response types reflect the way a person will respond, in terms of disease outcome, to an exposure by the end of a defined observation period. Since these response types describe the disease status of exposed individuals at the end of the observation period, they assess the excess exposure effect. To assess the etiologic exposure effect, we have adapted these response types (see endnote 3). Below, we describe three possible disease responses to an exposure of interest; *non-causal cases*, *etiologic cases *and *non-diseased*. The response type of an exposed individual who becomes diseased over the observation period is determined by his/her constellation of components at the moment of disease onset. In contrast, the response type of an exposed individual who does not become diseased over the observation period is determined by his or her constellation of components at the end of the observation period.

An exposed individual who first acquires a SC that does not include the exposure of interest is a non-causal case. The exposure has no effect on disease occurrence among non-causal cases; these individuals are assigned response type 1. An exposed individual who first acquires the causal partners of the exposure is an etiologic case. For etiologic cases, the exposure determines whether and when the disease occurs; these individuals are assigned response type 2. An exposed individual who does not acquire the causal partners of the exposure and does not acquire another SC that does not include the exposure is non-diseased. The exposure has no effect on disease occurrence among the non-diseased; these individuals are assigned response type 4 (see endnote 4).

For example, referring to Figure 2: Independent Mechanism, an exposed person (i.e. someone who has already acquired E) who then acquires U_2 _and U_3 _is a non-causal (type 1) case. An exposed person who then acquires U_1 _is an etiologic (type 2) case. And an exposed person who does not acquire U_1 _or both U_2 _and U_3 _is non-diseased (type 4).

The proportions of individuals in the population who are non-causal cases, etiologic cases and non-diseased are labeled as p1, p2 and p4, respectively (see Figure 3). The proportion of people who develop the disease under the condition of exposure is equal to the sum of the proportions of non-causal and etiologic cases, or p1+p2. The proportion of people who will develop the disease under the *etiologic counterfactual condition*, i.e., the proportion of exposed individuals who would have been diseased at their moment of disease onset had they not been exposed, is equal to the proportion of non-causal cases, or p1. However, the proportion of people in the same population that will develop the disease under the *excess counterfactual condition*, i.e. the proportion of exposed that would have been diseased at the end of the study had they not been exposed, includes both: (1) the proportion of non-causal cases, and (2) the subset of the etiologic cases who are etiologic redundant cases. As we will later show, the proportion of etiologic redundant cases is directly related to the extent to which redundancy obscures causal identification.

To identify this subset of the etiologic fraction, we must further specify these response types by distinguishing between redundant and non-redundant cases. We will refer to a person who acquires only one type of SC of disease (i.e. SCs that include the exposure or SCs that do not include the exposure, but not both types) over the study period as "non-redundant". We will refer to a person who acquires both types of SC (where at least one SC includes the exposure and one does not) as "redundant". Among the non-causal cases, we define a person who only acquires a SC(s) that does not include the exposure as a type 1^n^, where '1^n^' refers to non-causal, non-redundant. Individuals who are type 1^n ^develop the disease from a SC that does not include the exposure, and do not later acquire a SC that includes the exposure. The proportion of individuals who are non-causal, non-redundant over the study period is designated p1^n^. A non-causal case that acquires both types of SC(s) is labeled a type 1^r^, where '1^r^' refers to non-causal, redundant. Individuals who are type 1^r ^develop the disease from a SC that does not include the exposure but later acquire another SC that does include the exposure. The proportion of individuals who are non-causal, redundant over the study period is designated p1^r^.

Likewise, among the etiologic cases, we define a person who only acquires a SC(s) that includes the exposure as a type 2^n^, where '2^n^' refers to etiologic, non-redundant. Individuals who are type 2^n ^develop the disease from a SC that includes the exposure, and do not later acquire a SC that does not include the exposure (i.e. type 2^n ^individuals are excess cases). The proportion of individuals who are etiologic, non-redundant over the study period is designated p2^n^. An etiologic case that acquires both types of SC(s) is labeled a type 2^r^, where '2^r^' refers to etiologic, redundant. Individuals who are type 2^r ^develop the disease from a SC that includes the exposure but later acquire a SC that does not include the exposure. The proportion of individuals who are etiologic, redundant over the study period is designated p2^r^. Each of these specific response types, and their contribution to the proportion of exposed diseased individuals are labeled in Figure 3.

### Relationship between relative etiologic effect and relative excess effect

Using these proportions, we can calculate the relative etiologic and excess causal contrasts. The numerator of both RR_etiology _and RR_excess _is the proportion of the exposed who develop the disease by the end of the study period; (p1+p2) or (p1^n^+p1^r^+p2^n^+p2^r^). However, the denominators of RR_etiology _and RR_excess _differ. The denominator of RR_etiology _includes those individuals who would have developed the disease at the moment they did even in the absence of the exposure; p1 or (p1^n^+p1^r^). The calculation of RR_etiology _in terms of these specific response types is shown in Equation 1.(1)

The denominator of RR_excess _includes all individual who would have developed the disease by the end of the study in the absence of the exposure. The denominator of RR_excess _includes: (i) those individuals who, in the absence of the exposure, would have developed the disease at the moment they did; p1 or (p1^n^+p1^r^), and (ii) those individuals who would have developed the disease later over the study period; p2^r^. Thus, the denominator of RR_excess _is (p1^n^+p1^r^+ p2^r^). The calculation of RR_excess _in terms of these specific response types is shown below in Equation 2.(2)

Thus, the only difference between RR_etiology _and RR_excess _is p2^r ^in the denominator of RR_excess_. The relationship between these causal contrasts can be expressed as the ratio of RR_excess _to RR_etiology_, which reduces to Equation 3.(3)

As Equation 3 shows, RR_excess _underestimates RR_etiology _to the extent that there are individuals who are etiologic redundant (i.e., type 2^r^) in the population. As p2^r ^increases relative to p1, the discrepancy between RR_etiology _and RR_excess _increases. As discussed earlier, p2^r ^(and p1^r^, though it has no effect on excess versus etiology) will be larger as the prevalence of the components increases and/or when redundancy arises from dependent processes.

Here, we used the disease type notation to express the excess and etiologic causal effects in a population of people actually exposed. It is also possible to express redundancy in a general way for target populations of any size with varying exposure distribution (assuming exposure is randomly distributed) and all forms of disease frequency measures. Using the notation in Maldonado's and Greenland's paper "Estimating Causal Effects" (2002) [23], we provide an alternative expression of redundancy in Additional File 1.

In summary, the difference between RR_excess _and RR_etiology _is the extent that there are redundant, etiologic cases in the population; the magnitude of this difference is driven by the structure of the SCC model and the prevalence of its components. Given that both RR_excess _and RR_etiology _are both causal contrasts, there are two possible counterfactuals for the exposed. In addition to specifying whether the causal effect for the exposed, the unexposed or the entire population is of interest, epidemiologists should also specify whether the excess or etiologic causal effect is of interest. Because we are inherently interested in identifying causes, we conceptualize the excess effect as an underestimate of the etiologic effect. In the illustrative example below, we demonstrate the extent to which RR_excess _can underestimate RR_etiology_.

### Numeric example: A hypothetical study of 200 people

**Figure 4:**
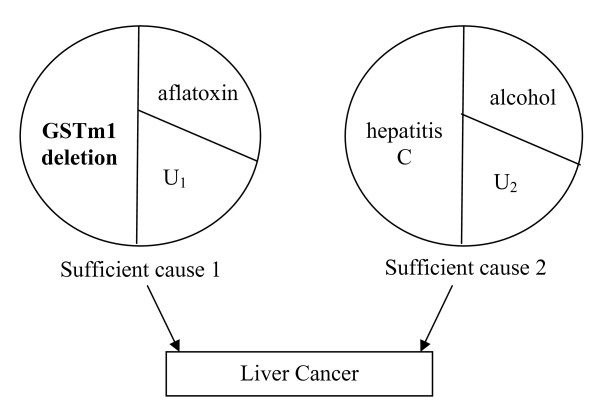
**Illustrative hypothetical example: two sufficient causes of liver cancer**.

To make the remaining discussion less abstract, we use the simplistic hypothetical causal model for liver cancer shown in Figure 4. In this hypothetical causal model, there are six dichotomous causal components that make up two SCs of disease. Supposing these are the only causes of liver cancer, an individual can either get liver cancer from exposure to: (1) a metabolic polymorphism of the glutathione-S-transferase gene (the GSTm1 deletion), aflatoxin and U_1_, or (2) hepatitis C infection, alcohol and U_2_.

An exposed individual (i.e. a person with the GSTm1 deletion) who acquires hepatitis C, alcohol and U_2 _before acquiring aflatoxin and U_1_, is a non-causal (type 1) case. An exposed individual who acquires aflatoxin and U_1 _before acquiring hepatitis C, alcohol and U_2 _is an etiologic (type 2) case. An exposed individual who does not acquire both aflatoxin and U_1 _and does not acquire hepatitis C, alcohol and U_2 _is non-diseased (type 4).

Imagine a researcher began with 100 pairs of individuals who were identical except for their GSTm1 status; 100 individuals had the GSTm1 deletion and 100 did not. The individuals with the deletion were exposed, and the individuals with normal GSTm1 were their counterfactuals. Furthermore, (for simplicity) imagine that all 200 individuals had components U_1 _and U_2 _at the start of observation.

Since the researcher did not know when the individuals will develop liver cancer (i.e. the induction period is unknown), she decided to follow the study population for 12 years. Imagine that liver cancer is immediately apparent and the researcher had a perfect diagnostic test and so did not need to account for a latency period. By the end of the study period, five of 100 exposed and 3 of 100 unexposed individuals were diseased. The risk of disease among the exposed by the end of the 12-year period was 0.05 (5/100), the risk of disease among the unexposed by the end of the same time period was 0.03 (3/100), and observed risk ratio (RR_observed_) was 1.7 (0.05/0.03).

**Figure 5:**
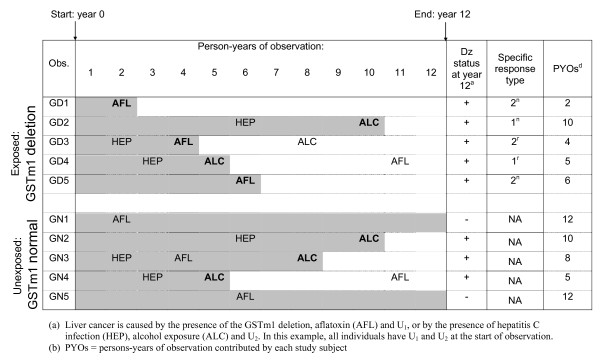
**Acquisition of components and disease (Dz) status for liver cancer (based on the hypothetical causal model shown in Figure 4) in ten individuals over the course of a 12 year study**.

Figure 5 displays the component acquisition and disease experience of the exposed individuals who developed liver cancer over the 12-year study period (labeled GD1 through GD5) and their unexposed counterparts (labeled GN1 through GN5). In addition to the 10 individuals shown in Figure 5, the study included 95 exposed people who remained non-diseased and their 95 unexposed counterparts who also remained non-diseased. For diseased individuals, the last component needed to first complete a SC is shown in bold font. In addition, each individual's disease status at the end of the study and specific response type are shown.

Assigning specific response types requires we examine the moment that each exposed individual developed the disease. If we examine the bolded factors in Figure 5, we can identify the moment of disease onset of each exposed individual (GD1 through GD5). Persons GD2 and GD4 were non-causal cases (types 1n and 1r, respectively), GD1 and GD5 were excess cases (type 2n) and GD3 was an etiologic redundant case (type 2r). Therefore, p1 was 0.02 (2/100), p2^n ^was 0.02 (2/100) and p2^r ^was 0.01 (1/100). RR_excess _is 1.7 [(0.02+0.02+0.01)/(0.02+0.01)]. Since the unexposed group represents the counterfactuals for the exposed group (i.e. there is no confounding or bias), RR_observed _is equal to RR_excess_.

RR_etiology _is 2.5 [(0.02+0.02+0.01)/0.02]. Persons GD3 and GN3 are the only pair in which the disease status of the unexposed counterpart at year 12 is not equivalent to his/her disease status at the moment the exposed person became diseased. From the moment that GD3 and GN3 were exposed to alcohol, the SCs for GD3 were redundant and the counterpart, GN3, became diseased from SC2. At this moment, GN3 was no longer an appropriate comparison for GD3. As a result, RR_etiology _was underestimated by RR_excess_.

Figure 5 also shows that there was also redundancy of SCs for GD4. After developing the disease from SC 2, GD4 also acquired aflatoxin, fulfilling SC 1. In this circumstance, the disease status of the counterpart (GN4) at the end of the study was equivalent to the disease status he or she had at the moment GD4 became diseased. Therefore, GN4 was an appropriate comparison for GD4, and the relationship between RR_excess _and RR_etiology _was unaffected by this pair.

The researcher could correctly conclude that the GSTm1 deletion yielded a relative excess risk of disease of 1.7. However, she could not determine the full etiologic effect of the deletion. Using Equation 3, we can see that the researcher captured 67% [0.02/(0.02 + 0.01) = 0.67] of the relative etiologic effect, or conversely that she missed 33%.

In this example, one etiologic redundant case among the 100 exposed people (person GD3) resulted in an appreciable underestimation of the etiologic effect. Clearly, the underestimation would have been more severe had there been more etiologic redundant cases. For instance, if GD1 had also been an etiologic redundant case, the etiologic effect would remain the same, while the observed RR would have been 1.25 [(5/100)/(4/100) = 1.25]. In this case, the researcher would have missed 50% [1-(0.02/(0.02 + 0.02)) = 0.50] of the relative etiologic effect.

Even in the absence of confounding and bias, our studies underestimate the etiologic effect to the extent that there are etiologic redundant cases in the study population by the end of the study period. Because the presence of redundancy is related to when we compare exposed and unexposed people, it may seem intuitive that we may be able to avoid or correct this underestimation. In discussing the problem of redundancy with colleagues, we find that epidemiologists often believe that redundancy is a latency issue, or can be resolved using matching and/or rate-based measures. However, as we discuss below, this is not the case.

## Can redundancy be avoided or resolved?

### The relationship between latency and redundancy

Redundancy occurs because there is a gap between the onset of disease in an exposed individual and when we compare the disease statuses of exposed and unexposed individuals. This gap allows exposed individuals to acquire more than one SC of disease, implying that minimizing this gap can reduce or avoid redundancy.

To see the causal effect of the exposure, we must compare the disease status of exposed and unexposed individuals after the disease is detectable in the exposed. When the disease event is detectable at onset (e.g. severe myocardial infarction, death, etc.), we theoretically can avoid redundancy by comparing the disease status of perfectly matched exposed and unexposed people at the moment of disease onset in the exposed (even if retrospectively). When the disease event is not detectable at onset (e.g. cancer, depression, etc.), we can avoid redundancy by comparing the disease status of an exposed-unexposed pair: (1) before they acquire another cause (i.e. before redundancy occurs), or (2) after they acquire another cause (i.e. after redundancy occurs) but before the disease is detectable in the unexposed. The full causal effect can be identified if we make our comparison after the latency period (i.e. the period after onset until the disease is detectable) ends in the exposed, but before the latency period ends in the unexposed.

Whether or not there is a latency period, redundancy can theoretically be avoided by comparing the disease status of a perfectly matched exposed and unexposed pair at the moment the disease was detectable in the exposed. (i.e. the moment of disease onset if there were no latency period). Even if it was possible to diagnose every individual at the moment the disease was detectable, our current effect measures cannot accommodate this information. Rather, our measures compare the disease status of groups of exposed and unexposed by the end of the study period. As shown above, even when only one exposed individual is an etiologic redundant case, the etiologic effect is underestimated.

### Matching

To minimize confounding and bias, epidemiologists employ various techniques to balance risk factors across the exposed and unexposed groups. One way to do this is to match exposed and unexposed individuals on other causes of disease that we think are imbalanced across the exposed and unexposed groups (i.e. suspected confounders). If all confounders are captured, matching avoids confounding. However, redundancy is a more subtle issue. To avoid redundancy, we would need to: 1) match exposed and unexposed individuals on all known and unknown risk factors, including those imbalanced (i.e. confounders) and those balanced (i.e. non-confounding causes of disease) across exposure groups, 2) match the individuals on the timing of acquisition of those risk factors, and 3) remove the unexposed counterpart for each individual from the population at risk at the moment each exposed person becomes diseased. Clearly, this is not within the realm of our current methods.

### Rate-based comparisons

As explained above, risk-based measures reflect disease occurrence by the end of the study. Since rate-based measures take into account when a person is diagnosed, it may seem intuitive that rate-based measures would be immune to the effects of redundancy. However, rates do not entirely resolve redundancy.

Unlike risk-based comparisons, rate-based comparisons are concerned with what happens to a group of people over time. In the absence of confounding and bias, rate-based measures indicate when an exposure causes individuals to develop disease earlier than they would have otherwise. However, rate-based measures do not validly estimate the etiologic effect. Under some circumstances when risk-based measures underestimate the etiologic effect of an exposure, rate-based measures can better estimate the full etiologic effect. Under other circumstances, rate-based measures can also materially underestimate the etiologic effect.

The observed rate of disease in a group of exposed people is the number of diseased (D) as a proportion of the exposed person-time at risk (PT_E_) by the end of a specified time period [p(D | PT_E_)] [22,24]. The observed rate of disease in a group of unexposed people is the number of diseased (D) as a proportion of the unexposed person-time at risk (PT_e_) by the end of the same time period [p(D | PT_e_)] [22,24]. We refer to the observed rate ratio as IR_observed _(see endnote 5). The calculation of IR_observed _is shown in Equation 4 [22,24].(4)

We can use the previous numerical example to investigate the utility of rates in estimating the etiologic effect when redundancy occurs (see Figure 5). Rather than waiting until the end of year 12, imagine the researcher tested each individual for liver cancer at the end of each year and thus knew the year each person became diseased. The person-time at risk for each individual (defined here as the number of years including the year of disease onset that each person was disease-free) is shaded and is expressed numerically as person-years of observation (PYOs).

There were 1,167 PYOs among the exposed (GSTm1 deletion); 27 PYOs among those shown in Figure 5 and 1,140 PYOs among the remaining 95 exposed individuals (95 people * 12 years = 1,140). There were 1,187 PYOs among the unexposed (GSTm1 normal); 47 PYOs among those shown in Figure 5 and 1,140 PYOs among the remaining 95 unexposed individuals. The rate of disease among the exposed by the end of the 12-year period was 0.0043 per person-year (5/1,167) and the rate of disease among the unexposed by the end of the study was 0.0025 per person-year (3/1,187). By multiplying by 12, the number of years of follow-up, we can express IR_observed _in the same units of time as the causal contrasts. Therefore, the IR_observed _was 1.7 per 12 person-years [(0.0043*12)/(0.0025*12)].

Despite the fact that: (1) this is the best-case scenario in which there is no confounding or bias, and (2) rates take into account when each individual became diseased - the researcher cannot determine the full etiologic effect of the deletion. As with risk-based measures, rate-based measures do not compare the disease status of perfectly matched pairs of exposed individuals and their unexposed counterparts at the moment the exposed are diagnosed. As a result, when redundancy occurs, the number of diseased in the unexposed at the end of the study is greater then the number of diseased in the exposed if the exposure had been absent at the moment of disease onset.

Rate-based measures can only precisely estimate the etiologic effect under the same conditions as risk-based measures; after the exposed person becomes diseased, the unexposed person should not contribute to either the number of diseased or the person-time among the unexposed group. Thus, the total person-time in the exposed and unexposed groups would cancel out and the "rate ratio" would be equivalent to the etiologic effect (if the person-time cancels out, the rate ratio becomes a risk ratio). Although incorporating person-time makes it apparent when there are some people who get disease earlier if exposed (which tells us that the exposure has some etiologic effect), it does not resolve the fact that the number of diseased within the unexposed is an overestimation of the number of diseased within the counterfactual group. In fact, in their 1988 paper, Greenland and Robins demonstrate that the incidence density fraction (or, rate fraction) is not necessarily equivalent to either the etiologic or excess fractions [3]. The same is true for the rate ratio.

In summary, estimation of the etiologic effect of an exposure requires that the disease status of exposed individuals be compared with the disease status of their unexposed counterparts at the moment the disease is detectable in the exposed. This necessitates that: (1) each unexposed person provides a perfect proxy for the counterfactual disease risk in his/her exposed counterpart, (2) we watch people over time and assess their disease status soon after onset (or soon after the disease is detectable), (3) that our measures use this information to compare exposed persons to their unexposed counterparts at the moment of diagnosis in the exposed, and 4) we remove the unexposed person from the population at risk when the exposed counterpart becomes diseased. Our current measures do not do this; even if conditions 1 and 2 are met, the disease status of a group of exposed people is compared with that of a group of unexposed people by the end of the study period (condition 3 is not met). Furthermore, the unexposed counterparts are not removed from the population at risk when the exposed are diagnosed (condition 4 is also not met). As a result, both risk- and rate-based measures are affected by redundancy.

## Conclusion

Redundancy, the acquisition of multiple sufficient causes of disease by an individual over a given observation period, causes a discrepancy between the excess and full etiologic effects of a cause. Even in the absence of confounding and bias, the observed risk ratio represents only a subset of the full etiologic effect. The proportion of redundant individuals among etiologic cases is reflected in the discrepancy between the etiologic effect necessary to identify causes and the excess effect we are able to estimate. For an individual, redundancy could be avoided if we compared the exposed person to his/her unexposed counterpart precisely at that moment disease was detectable. However, our methods, including rate-based measures, do not allow us to compare perfectly matched pairs of exposed and unexposed individuals. In other words, epidemiology cannot find the causal effect for an individual; it can only find the average causal effect across individuals. Thus, even in the best-case scenario where we know the moment of disease onset for each individual in a study, we cannot precisely estimate the full causal effect. Even if precise estimation is not our goal, to the extent that redundancy exists, we may incorrectly conclude that the exposure is not causal.

Our liver cancer example demonstrates how causal identification can be impeded when there is redundancy of sufficient causes with mutually exclusive and independent components (i.e. redundancy arising from an Independent Mechanism). This scenario is illustrated in Figure 4, where none of the sufficient causes share any components and there are no associations between any components. This illustrates that even in the simplest scenarios, redundancy can influence our effect estimates. However, there are more complex scenarios in which redundancy may be more profound. In fact, the underestimation will be worse under any causal model in which the proportion of etiologic redundant cases relative to the baseline risk increases. For instance, under a causal model with many sufficient causes that do not include the component of interest, there will be more individuals with redundant response types (both etiologic and non-causal redundant cases) because they are more likely to acquire more than 1 sufficient cause by chance. The proportion of redundant individuals will also increase if a causal partner of the exposure of interest also participates in a sufficient cause that does not include the exposure. In addition, if there is ordering of a causal partner and a component in another sufficient cause in time (e.g. people who acquire both causes are more likely to be etiologic redundant cases than non-causal redundant cases), the impact of redundancy may be more severe.

Epidemiologists consider an effect estimate for the exposed to be valid if the risk of disease in the unexposed group is equal to the risk of disease from other causes in the exposed. Nonetheless, valid effect estimates may still underestimate the full etiologic effect of an exposure. To ensure that we are measuring the full effect of an exposure, either redundancy must not exist, or we must develop new methods to account for the impact of redundancy. This problem is not isolated to observational epidemiology. Even in a randomized trial, where it is more likely that confounding and bias are absent, redundancy can impede causal identification.

Furthermore, redundancy may prevent us from identifying effective interventions. Imagine we want to reduce the burden of liver cancer in some population. Suppose that (based on the causal model in Figure 4) sufficient cause 1 is much more prevalent (i.e. a much more important cause of the disease) in this population than sufficient cause 2, and sufficient cause 1 is almost always completed first. This might be the case in a population that is exposed to dietary aflatoxin during childhood, but is exposed to hepatitis C and alcohol exposure in later years. Imagine we conduct a study to determine whether aflatoxin (an exposure on which we can intervene) is a cause of disease in this population, but we miss its causal effect or regard it as unimportant due to redundancy. Then in a later study, we identify alcohol as cause of liver cancer and decide to reduce the proportion exposed to alcohol. However, our intervention is unsuccessful - the prevalence of liver cancer is nearly as high as before we intervened since we have not eliminated the more important (and earlier onset) cause, sufficient cause 1.

The consequences of redundancy may be profound. In subsequent papers, we will examine the circumstances under which redundancy is likely to impede causal identification and quantify the impact on effect estimates using simulated data. Other next steps include characterizing the impact of redundant causation in the presence of preventive effects (i.e. the situation in which the exposure of interest is simultaneously causative and preventive), and understanding the impact of redundant causation in the context of confounding or bias.

## Competing interests

The authors declare that they have no competing interests.

## Endnotes

1. We use to the term "acquires" to mean "is or becomes exposed to" a component cause in an SCC model. We chose this term to avoid confusion between "being exposed" (i.e. having the exposure of interest) and having or getting another "exposure" (i.e. another causal component).

2. Under the SSC model depicted in Figure 2: Independent Mechanism, it is theoretically possible that, by chance, an individual simultaneously acquires the components of more than 1 SC, resulting in overdetermination.

3. Our adaptation of Greenland and Robin's [9] original response type schema further specifies the response types to reflect how and when a person becomes diseased, in addition to whether s/he becomes diseased over a specified time period under the condition of being exposed or unexposed.

4. Our response type schema includes response types 1, 2 and 4 but excludes response type 3 for consistency with the response type schema developed by Greenland and Robins [9]. Response type 3 is reserved to describe individuals who acquire all of the other components of a sufficient cause that requires the absence of the exposure of interest to cause disease, such that if *not exposed *they get the disease from that sufficient cause. Although for many diseases all four response types are reasonable, for simplicity, our work only considers causal scenarios in which the exposure is not simultaneously causal and preventive.

5. We use the letter "I" to indicate when a measure is based on rates because the rate is also referred to as the incidence density.

## Authors' contributions

This research was part of NG's dissertation. NG conceived the idea and drafted the manuscript. UB provided critical intellectual content through discussions and revision of the manuscript. Both authors read and approved the final manuscript.

## Supplementary Material

Additional file 1 **Alternative expression of etiologic and excess causal effects**. Click here for file
