# Supplementary material for: Redundant causation from a sufficient cause perspective
Source: Epidemiol Perspect Innov. 2010 Aug 2;7:5. doi: 10.1186/1742-5573-7-5 (PMC2922199; doi:10.1186/1742-5573-7-5)
Supplement: Additional file 1 — Alternative expression of etiologic and excess causal effects. [file 1742-5573-7-5-S1.DOC]

**Additional file 1**

**Alternative expression of etiologic and excess causal effects**

In this paper, we have adapted the disease response type notation to frame the issue of redundancy. It is possible to express redundancy in a general way for target populations of any size with varying exposure distribution (assuming exposure is randomly distributed) and all forms of disease frequency measures. For example, we can expand the notation of Maldonado and Greenland (2002) “Estimating Causal Effects” [23] to alternatively express redundancy:

A_11_

A_10_

B_1_

A_01_

A_00_

B_0_

Target if exposure distribution 1

Target if exposure distribution 0

A_1_

A_0_

where A_i_ is the number of cases that would occur in the target under exposure distribution i, A_i1_ is the number of etiologic cases under exposure distribution i, A_i0_ is the number of non-causal cases under exposure distribution i, and B_i_ is the number of people in the target population under exposure distribution i.

Then,

(A_1_ / B_1_)

RR_excess_ =

Eq. A1

(A_0_ / B_0_)

(A_1_ / B_1_)

RR_etiology_ =

Eq. A2

(A_00_ / B_0_)

RR_excess_ / RR_etiology_  =

Eq. A3

(A_00_)

(A_0_)

RR_excess_ is the causal relative risk of any form (i.e. incidence-proportion ratio, odds ratio, or person-time incidence rate ratio) that measures the excess causal effect of exposure; in Maldonado and Greenland’s terminology, this is RR_causal_ [23]. RR_etiology_ is the causal relative risk of any form that measures the full causal effect of exposure.

The relationship between these causal contrasts can be expressed as the ratio of RR_excess_ to RR_etiology_, which reduces to Equation A3.

Note that the disease type notation presented in the main body of the paper assumes that we are interested in estimating the excess and etiologic causal effects in a population (of any size) of people actually exposed (i.e. the comparison is the counterfactual; the same population, all else equal, had they not been exposed). Thus, the disease type equations presented in the paper for RR_etiology_  (Eq. 1), RR_excess_ (Eq. 2), and the ratio of RR_excess_ to RR_etiology_ (Eq. 3) are only equivalent to equations A1-A3 if exposure distribution 0 is 0%.

Then,

RR_excess_ =

(p1^n^ + p1^r^ + p2^n^ + p2^r^)

(p1^n^ + p1^r^ + p2^r^)

Eq. A4

= (A_1_ / B_1_)

(A_0_ / B_0_)

RR_etiology_ =

(p1^n^ + p1^r^ + p2^n^ + p2^r^)

Eq. A5

(p1^n^ + p1^r^)

= (A_1_ / B_1_)

(A_00_ / B_0_)

p1

(p1 + p2^r^)

RR_excess_ / RR_etiology_  =

Eq. A6

p1^n^ + p1^r^

(p1^n^ + p1^r^ + p2^r^)

=

= (A_00_)

(A_0_)

In terms of disease response types, Equation A6 shows that RR_excess_ underestimates RR_etiology_ to the extent that there are individuals who are etiologic redundant (i.e., type 2^r^) in the population. Alternatively, Equation A6 shows that RR_excess_ underestimates RR_etiology_ to the extent that there are causal cases in population A_0_. The relationship between these alternative notations when exposure distribution 0 is 0% is shown in Figure A1 below.

**Excess fraction = p^k^n**

**Redundant fraction = p^k^r**

**Etiologic cases = A_i,1_**

**Etiologic fraction = p2**

##### Non-diseased

**Excess cases**

**[2n]**

**[1n]**

**[1r]**

**Etiologic redundant cases**

**[2r]**

**[4]**

**Non-causal fraction = p1**

**Non-causal cases = A_i,0_**

**p4**

**Number of people in target population under exposure distribution i = B_i_**

Figure A1. Relationship among etiologic, excess, etiologic redundant cases and non-causal cases. Where, ‘i’ represents the exposure distributions (1 or 0), ‘k’ represents etiologic or non-causal (2 or 1), ‘n’ equals non-redundant, ‘r’ equals redundant, ‘p’ means proportion

Such that,

B_i_ is the number of people in the target population under exposure distribution i

A_i_ is the number of cases in the target population under exposure distribution i

A_i,1_ is the number of etiologic cases in the target population under exposure distribution i

A_i,0_ is the number of non-causal cases in the target population under exposure distribution i
